# Simulation of Enzyme Catalysis in Calcium Alginate Beads

**DOI:** 10.1155/2012/459190

**Published:** 2012-10-31

**Authors:** Ameel M. R. Al-Mayah

**Affiliations:** Biochemical Engineering Department, Al-Kawarizimi College of Engineering, University of Baghdad, Baghdad, Iraq

## Abstract

A general mathematical model for a fixed bed immobilized enzyme reactor was developed to simulate the process of diffusion and reaction inside the biocatalyst particle. The modeling and simulation of starch hydrolysis using immobilized **α**-amylase were used as a model for this study. Corn starch hydrolysis was carried out at a constant pH of 5.5 and temperature of 50°C. The substrate flow rate was ranging from 0.2 to 5.0 mL/min, substrate initial concentrations 1 to 100 g/L. **α**-amylase was immobilized on to calcium alginate hydrogel beads of 2 mm average diameter. In this work Michaelis-Menten kinetics have been considered. The effect of substrate flow rate (i.e., residence time) and initial concentration on intraparticle diffusion have been taken into consideration. The performance of the system is found to be affected by the substrate flow rate and initial concentrations. The reaction is controlled by the reaction rate. The model equation was a nonlinear second order differential equation simulated based on the experimental data for steady state condition. The simulation was achieved numerically using FINITE ELEMENTS in MATLAB software package. The simulated results give satisfactory results for substrate and product concentration profiles within the biocatalyst bead.

## 1. Introduction 

Enzyme immobilization on to supports (or carriers) and their applications as catalysts have grown considerably during the last three decades, and during the last few years have become the most exciting aspects of biotechnology [1–3]. Several methods of enzyme immobilization exist and can be classified into three main categories: carrier binding, cross linking, and entrapment [1]. A number of advantages of enzyme immobilization on to support and several major reasons are the ability to stop the reaction rapidly by removing the enzyme from the reaction solution (or vice versa), products being free of enzyme (especially useful in the food and pharmaceutical industries), reduced effluent disposal problems, suitability for continuous reactor operation, and multiple or respective use of a single batch of enzymes, especially if the enzymes are scarce or expensive, their applicability to continuous processes, and the minimization of pH and substrate-inhibition effects. This has an obvious economic impact and allows the utilization of reactors with high enzyme loads [4].

Enzyme entrapment within a gel matrix is one of the enzyme immobilization ways. In this way, the enzyme is surrounded by a semipermeable membrane. Enzyme support of a specific structure permits the contact between the substrate and the biocatalyst in an appropriate way [5, 6]. 

Enzyme entrapment in calcium alginate beads has been shown to be a relatively simple and safe technique [7–9]. Calcium alginate beads made with 2% (w/v) solution have an average pore diameter of 80 to 100 Å [6, 7, 10]. Starch molecules are very large, often reaching a molecular weight of 80 million Daltons [9, 11]. It is expected that starch hydrolysis reaction occurs more effectively if enzyme is bound to the surface. Several methodshave been compared and reported for the immobilization of *α*-amylase on different supports which provide useful information on the efficiency of the hydrolysis of starch into smaller sugars [12–15].

The catalytic activity of the immobilized enzyme is affected mainly by the limitations of internal and external mass transfer. External mass transfer limitations can be reduced by changing the reactor hydraulic conditions (e.g., the level of agitation), while the internal mass transfer limitations are severe and much more difficult to solve [4]. Enzyme biochemical properties and reaction type and kinetics as well as support chemical and mechanical properties all affect the internal mass transfer [4].

To overcome these limitations, small carrier particle size is proposed to be used. Use of small particle size complicates the reactor operation due to the increasing the pressure drop (i.e., in case of packed bed reactor) or increasing catalyst washout (i.e., fluidized bed reactors). However the idea cannot be generalized for every enzyme. For example, in the case of enzymes that are inhibited by substrate concentration, the operation can be improved by the reduction of the local substrate concentration. 

Many mathematical models have been developed by a large number of researchers supported by experimental data, for different types of reactors and mode of operation containing immobilized enzymes [16–19]. Simulation of these models can contribute to improve the understanding of the immobilized systems as well as in the prediction of substrate consumption and product formation rates.

Some of theses models consider the mass transfer of substrate from the bulk to the active sites where the enzyme is immobilized inside the carrier where the reaction takes place. Fick's law is used to model the mass transfer inside the biocatalyst particle [17, 20]. Enzyme catalysis is nonlinear which makes mathematical models more complex, even for the simple Michaelis-Menten kinetics model [1, 20–22].

The present study aimed to simulate the process of diffusion and reaction inside the biocatalyst particle. A set of differential equations obtained for spherical immobilized biocatalyst particles allowings the determination of the concentration profile of substrate and product within the biocatalyst in terms of particle geometry (radius) and the concentration of substrate in the bulk liquid phase. 

The hydrolysis reaction of corn starch catalyzed by immobilized *α*-amylase within hydrogel matrix was chosen and a mathematical model including the effects of diffusion rate and reaction rate parameters in steady state conditions with scaling analysis were used in this study.

## 2. Kinetic Parameters

Starch is a major component in many crops such as wheat, maize, tapioca, corn, and potato [2]. Starch consists of a mixture of amylose (15–30%) and amylopectin (70–85%). Starch is hydrolyzed to low molecular weight hydrocarbons by the action of either acids or enzymes [23]. 

*α*-amylase enzyme hydrolyzes the internal *α*-1,4-glycosidic links that exist in amylase and amylopectin to produce low molecular weight products [23–25]. Starch hydrolysis is influenced by several factors such as crystal structure, particle size, amylase and amylopectin content, and the presence of enzyme inhibitors [2, 24]. The kinetics of starch hydrolysis follows the Michaelis-Menten model as confirmed by results of many authors [2, 24] and as explained by (1).

(1) $$\begin{array}{c} E+S\overset{k_{1}}{\underset{k_{-1}}{⇌}}ES\overset{k_{2}}{\to }E+P, \end{array}$$

where *E* is the enzyme concentration, *S* is the substrate concentration, *k*_1_ is the rate constant for the forward reaction between enzyme and substrate, *k*_−1_ is the rate constant for the backward reaction, and *k*_2_ denote the rate constant for the ES complex dissociation.

The rate of reactant consumption or product formation can be expressed as [26–28]:

(2) $$\begin{array}{c} v=V_{\max}\frac{\left[S\right]}{K_{m}+\left[S\right]}=k_{2}{\left[E\right]}_{0}\frac{\left[S\right]}{K_{m}+\left[S\right]}, \end{array}$$

where

(3) $$\begin{array}{c} K_{m}=\frac{k_{-1}+k_{2}}{k_{1}}, \end{array}$$

(4) $$\begin{array}{c} V_{\max}=k_{2}{\left[E\right]}_{0}. \end{array}$$

[*E*]_0_ denoted the initial enzyme concentration.

For plug flow reactor, (2) can be rewritten as follows [28–30]:

(5) $$\begin{array}{c} -\frac{d\left[S\right]}{dt}=V_{\max}\frac{\left[S\right]}{K_{m}+\left[S\right]}, \end{array}$$

where (−*d*[*S*]/*dt*) represent the rate of disappearance of substrate.

Rearranging (5) and integration for the boundary conditions [*S*]  =  [*S*_0_] at *t* = 0 and [*S*]  =  [*S*_*t*_] at time *t*,

(6) $$\begin{array}{c} -K_{m}\int_{S_{0}}^{S}\frac{d\left[S\right]}{\left[S\right]}-\int_{S_{0}}^{S}d\left[S\right]=V_{\max}\int_{0}^{t}dt \end{array}$$

yields the integrated form of the Michaelis-Menten model:

(7) $$\begin{array}{c} K_{m}\ln\frac{\left[S_{0}\right]}{\left[S_{t}\right]}+\left[S_{0}\right]-\left[S_{t}\right]=V_{\max}t. \end{array}$$

For fixed bed reactor, the reaction time or the residence time the reactant spends in the reactor is equal to *V*_*R*_/*Q*, where *V*_*R*_, is the reactor volume and *Q* is the substrate volumetric flow rate. Reactor voidage can be expressed as *ε* = *V*_*b*_/*V*_*R*_ (where *V*_*b*_ denoted the volume of the immobilized enzyme beads), then the residence time can be expressed as *t* = *V*_*b*_/*Q* · *ε*.

Rearranging (7) yields

(8) $$\begin{array}{c} V_{\max}=\frac{K_{m}}{t}\ln\frac{\left[S_{0}\right]}{\left[S_{t}\right]}+\frac{\left[S_{0}\right]-\left[S_{t}\right]}{t}. \end{array}$$

Equation (8) can be further arranged in terms of conversion as:

(9) $$\begin{array}{c} \frac{\left[S_{0}\right]}{K_{m}}\frac{X}{t}=\frac{\ln\left(1-X\right)}{t}+\frac{V_{\max}}{K_{m}}, \end{array}$$

or

(10) $$\begin{array}{c} \frac{\ln\left(1-X\right)}{t}=\frac{V_{\max}}{K_{m}}-\left(\frac{\left[S_{0}\right]}{K_{m}}\right)\frac{X}{t}, \end{array}$$

where *X* = (([*S*_0_] − [*S*])/[*S*_0_]).

 A plot of *X*/*t* versus ln⁡(1 − *X*)/*t* gives a straight line with slope equal to (−[*S*_0_]/*K*_*m*_) and intercept of *V*_max⁡_/*K*_*m*_.

## 3. Mathematical Model 

In the present study, the enzyme was immobilized on alginate beads; the intraparticle mass transfer resistance can be affecting the rate of enzyme reaction. In order to derive an equation that shows the effect of mass-transfer on the effectiveness of immobilized enzyme. The following assumptions were made in developing and solving the mathematical model.

1. The reaction takes place at a constant temperature.
2. Enzyme concentration is constant and uniformly distributed within the beads.
3. Enzyme activity is uniform within the beads.
4. The pressure drop across the reactor and other mechanical effects are negligible.
5. All physical and transport properties are constant except rate constant.
6. Steady state conditions.
7. The substrate concentration is constant within the bulk liquid.
8. Enzyme kinetics is well described by the Michaelis-Menten model.
9. External mass transfer limitations are negligible.
10. The immobilized enzyme bead is spherical axis-symmetric geometry.
11. Constant diffusivities of substrate and products within the beads.
12. Mass transfer through the immobilized enzyme occurs via molecular diffusion.

**Figure 1:**
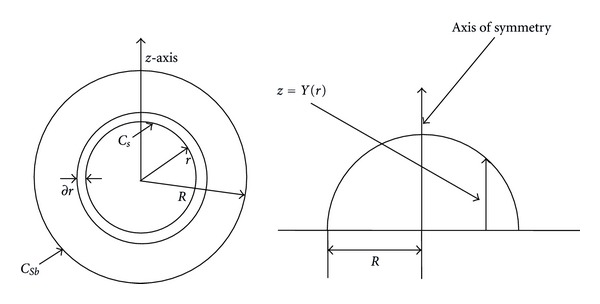
Shell balance for a substrate in an immobilized enzyme [29].

In this model cylindrical coordinates were used to locate the domain where diffusion and enzyme activity take place. Based on the above assumptions, the following unsteady state diffusion-reaction partial differential equations of mass balances for the product and substrate concentrations within the immobilized enzyme bead (and as shown in Figure 1) can be written as:

(11) $$\begin{array}{c} \text{Accumulation}=\text{Input}-\text{Output}+\text{Generation}, \end{array}$$

(12) $$\begin{array}{c} \frac{\partial S}{\partial t}=D_{s}\left(\frac{1}{r}\frac{\partial S}{\partial r}\left(r\frac{\partial S}{\partial r}\right)+\frac{\partial^{2}S}{{\partial z}^{2}}\right)-\frac{V_{\max}S}{K_{m}+S}, \end{array}$$

where *S* and *P* are denoted to the substrate and product concentration, *z* and *r* are the cylindrical coordinates, and *D*_*s*_ and *D*_*p*_ denoted the effective diffusivity of substrate and product.

The *z*-axis is the axis of symmetry of the enzyme bead that measures the distance from the planar bead. The variable *r* measures the radial distance from the *z*-axis. 

The initial and boundary conditions to be assumed for the present problem are as follows:

(13) P=S=0 at  t=0  for  z<Y(r),P=0 at  z=Y(r),S=S0 at  z=Y(r),(∂P∂z)=(∂S∂z)=0 at  z=0,(∂P∂r)=(∂S∂r)=0 at  r=0.

In order to simplify the solution of (12) the following dimensionless parameters were introduced here as [29]:

(14) Cs=SSo, Cp=PSo, τ=tR2/Ds,r˙=rR, z˙=zR, ϕ=R3Vmax⁡DsKm,β=SoKm, λ=DpDs Y−(r˙)=Y(r)R.

Equation (12) can be rewritten in the normalized form with the dimensionless parameters as:

(15) $$\begin{array}{c} \frac{\partial C_{s}}{\partial \tau }=\frac{1}{\dot{r}}\frac{\partial C_{s}}{\partial \dot{r}}\left(\dot{r}\frac{\partial C_{s}}{\partial \dot{r}}\right)+\frac{\partial^{2}C_{s}}{\partial {\dot{z}}^{2}}-\frac{9\phi^{2}C_{s}}{1+\beta C_{s}}, \end{array}$$

(16) $$\begin{array}{c} \frac{\partial C_{p}}{\partial \tau }=\lambda \left[\frac{1}{\dot{r}}\frac{\partial C_{p}}{\partial \dot{r}}\left(\dot{r}\frac{\partial C_{p}}{\partial \dot{r}}\right)+\frac{\partial^{2}C_{p}}{\partial {\dot{z}}^{2}}\right]+\frac{9\phi^{2}C_{s}}{1+\beta C_{s}}. \end{array}$$

The dimensionless parameter *ϕ* known as Thiele modulus is very important because it relates the reaction rate with diffusion rate. In order to simplify the calculations by relating the product concentration to substrate concentration (15) and (16) can be reduced to one linear partial differential as follows:

Let *W* = *C*_*s*_ + *C*_*p*_, then, for a steady state conditions, the change in substrate concentration, ∂*C*_*s*_/∂*τ*, and ∂*C*_*p*_/∂*τ* are equal to zero, then (15) will become:

(17) $$\begin{array}{c} \frac{1}{\dot{r}}\frac{\partial C_{s}}{\partial \dot{r}}\left(\dot{r}\frac{\partial C_{s}}{\partial \dot{r}}\right)+\frac{\partial^{2}C_{s}}{\partial {\dot{z}}^{2}}-\frac{9\phi^{2}C_{s}}{1+\beta C_{s}}=0. \end{array}$$

To simulate the reaction inside the biocatalyst, (17) and *W* = *C*_*s*_ + *C*_*p*_ are used in the present study with the following initial boundary conditions:

(18) W=Cs=0 at  τ=0,  z˙<Y−(r˙)W=Cs=1 at  z˙=Y−(r˙)∂Cs∂z˙=0 at  z˙=0∂Cs∂r˙=0 at  r˙=0.

## 4. Experimental Work

### 4.1. Materials

Sodium alginate salt (type Spectrum Chemical Mfg. Corp.), calcium chloride (CaCl_2_ *·*2H_2_O, of BDH type), fungal amylase powder (EC 3.2.1.1; 1,4-*α*-D-glucose glucanohydrolase, of MP Biomedicals type), corn starch (BDH), and HCl (BDH) were used in this study. All other chemicals were analytical grade reagents.

### 4.2. Buffer Solution

8 g of KH_2_PO_4_ (monobasic phosphate) and 0.2 g of K_2_HPO_4_ (dibasic phosphate) were dissolved in 0.5 liter of water to make a pH buffer solution in order to keep the pH value near to 5.5 during the enzymatic hydrolysis of the starch. 

### 4.3. Iodine Solution

100 mL of iodine solution (0.5% KI and 0.15% I_2_) was diluted with distilled water till the final volume reached 300 mL. It was used to make a complex compound by reaction with residual starch in the collected samples. The final complex compound has a deep blue color in order to measure the absorbency of this compound.

### 4.4. Preparation of Substrate Solution

40 g of corn starch powder was mixed with 50 mL of water in a beaker. The slurry was added to 900 mL of warm water in a large beaker. During this period, the slurry was mixed well using a magnetic stirrer and then cooled to room temperature to get the final gelatinized starch solution. Additional volume of water was added in order to bring the total volume to 1 liter.

A few drops of iodine were added to the solution, and the solution color changed to blue which indicates the presence of starch in the solution.

An equal volume of the above buffer and starch solutions were mixed. The resulting solution contains 20 g/L of starch in a buffered environment.

### 4.5. Enzyme Immobilization

3% (w/v) solution of sodium alginate was prepared by dissolving 15 g of it in 500 mL of water. During the preparation, sodium alginate powder was added slowly (to prevent clomping) to the beaker of water while stirring on a magnetic stirrer. After that, 0.01 g of *α*-amylase enzyme powder was mixed with 150 mL of sodium alginate solution. The final mixture was dropped using a syringe into 500 mL solution of 0.2 M CaCl_2_. Finally, the beads were left for 2 hours in the calcium solution to get the final hardened form of 2 mm average bead diameter. The final beads were removed from the calcium solution and washed five times with distilled water to remove the excess calcium chloride.

### 4.6. Immobilized Enzyme Assay

20 mL of 3% starch solution and 2 g of calcium alginate beads were assayed by using Bernfeld method [31]; also this method is recommended by Sigma-Aldrich Company [32]. Details of this test are stated in this reference. One unit was defined as the amount of amylase that produced 1 mmole of reducing sugar under assay condition per gram of bead.

### 4.7. Fixed Bed Reactor

**Figure 2:**
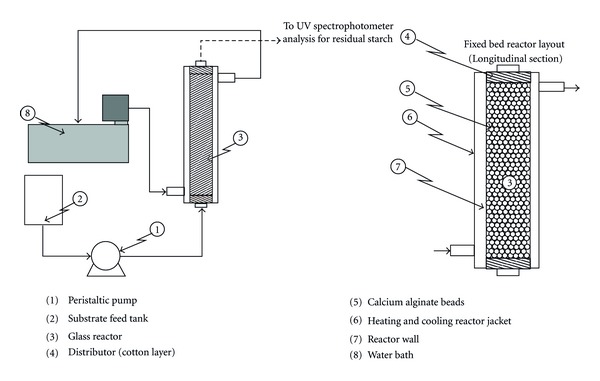
Experimental fixed bed reactor setup.

A 2 cm × 20 cm glass column was used in the present study as a fixed bed reactor. The reactor is surrounded with a jacket of water in order to keep the reaction in an isothermal condition. Two layers of cotton 2 mm thick were placed at the two ends of the column in order to supports the beads and distribute the substrate solution uniformly. The substrate flow rate was adjusted using a dosing pump (B. Braun Melsungen AG, model: 870602) and in a range of 0.2–5.0 mL/min. Samples of product effluents were collected at specific time intervals when the conditions were in steady state. In this study the hydrolysis of corn starch was carried out at constant temperature of 50°C, atmospheric pressure, and at constant pH of 5.5.  Figure 2 shows the experimental fixed bed reactor setup.

### 4.8. Packed Bed Void Fraction

The void fraction (*ε*) was determined experimentally using liquid impregnation method which can be illustrated as follows.

A 60 cm^3^ reactor volume was used in this work. First, the reactor was filled with the immobilized enzyme beads, and then distilled water was poured in it till it covers all the beads. After that the reactor was drained to measure the volume of water which is equal to the volume of the void. The void fraction can be calculated using the following equation:

(19) $$\begin{array}{c} \varepsilon =\frac{\text{Volume}\;\text{of}\;\text{voide}}{\text{Reactor}\;\text{volume}}. \end{array}$$

### 4.9. Enzyme Beads

Enzyme beads were placed in the reactor between two layers of cotton. The beads have an average diameter of 2 mm and density of 1.2 g/cm^3^. The packed bed has an average void fraction of 0.42.

### 4.10. Stopping Solution

0.1 N HCl solution was used as a stopping solution to stop the hydrolysis reaction of starch for the collected samples in order to analyze the starch content. 

### 4.11. Reactor Operational Efficiency

The entrapment efficiency was used to express the reactor operational efficiency. The entrapment efficiency was calculated at the same hydrolysis conditions. A buffer solution (pH 5.5) was used to wash the beads and the effect of feed flow rate (i.e., residence time) on the entrapment efficiency was determined in the range of 0.2–5 mL/min. The efficiency of entrapment was evaluated during 20 h continuous operation in a fixed bed reactor by collecting the samples from the reactor outlet solution at the end of 1, 2, 4, 6, 8, 10, 15, and 20 h, and calculating the unentrapped enzyme using the same procedure of enzyme assay. The entrapment efficiency of the enzyme was calculated using the following equation:

(20) $$\begin{array}{c} \text{Entrappment}\;\text{Efficiency}\;\left(\%\right)=\frac{C_{\text{en}}-C_{\text{un}}}{C_{\text{en}}}\times 100, \end{array}$$

where *C*_en_ is the enzyme initial amount and *C*_un_ is the enzyme amount in the outlet solution.

### 4.12. Starch and Products Analysis

The collected samples at timed intervals were analysed using Cintra 5 Double Beam UV-Spectrophotometer for residual starch content. Sample absorbency was measured at 620 nm.

## 5. Results and Discussion

### 5.1. Starch Hydrolysis

**Figure 3:**
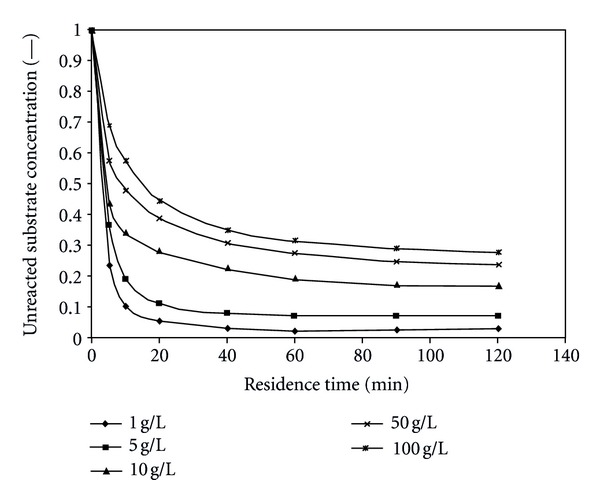
Effect of residence time on substrate concentration at different substrate initial concentrations (pH = 5.5, *T* = 50°C).

The effect of substrate (starch) flow rate on substrate concentration is explained in Figure 3. It can be observed that as the substrate flow rate increases substrate conversion decreases and at different values of substrate initial concentration, because the residence time is inversely proportional to substrate flow rate. Figure 3 shows the relation between residence time and substrate concentration. In this figure it can also be observed that the relation is linear at the first quarter of the time domain, and then the rate of change decreases rapidly. This is due to the fact that at the beginning of the reaction there is not a lot of substrate present near the enzymes, and the rate increases as the substrate increases because this will give the enzymes more substrate to work on; the rate of change continues till at a certain point, the rate of change decreases, because most of the active sites of enzyme within the bead will be saturated with the substrate to act on. After this point, the substrate concentration becomes too much for the enzyme to work on and the rate of change does not increase further so the rate of reaction becomes nearly constant.

### 5.2. Reactor Performance

**Figure 4:**
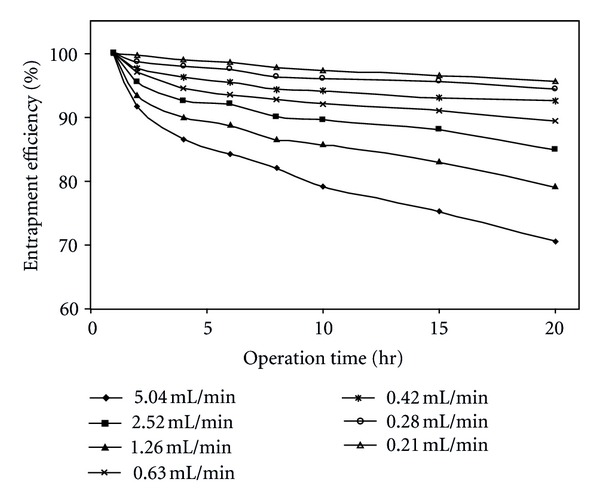
Effect of reactor operation time on entrapment efficiency at different feed flow rates (pH = 5.5, *T* = 50°C, [*E*]_0_ = 0.081 g/L).

Volumetric activity is another important parameter for bioreactor; it allows decreasing reactor volume and reduces production costs. In this section, the performance as stability of the entrapped *α*-amylase enzyme in the beads was evaluated in a continuous mode.  Figure 4 shows the relationship between the entrapment efficiency and feed flow rate (i.e., residence time). A low decrease in reactor stability with time of operation can be observed leading to a small change in reactor stability. Less than 5% reduction in the entrapment efficiency (95%) was observed at the end of 20 h continuous operation and at 0.21 mL/min feed flow rate. As the flow rate increases entrapment efficiency decreases and reached 70% at 5.04 mL/min feed flow rate and for the same operation. This can be attributed to the enzymatic leakage into the buffer solution from the alginate beads. As the reactor volumetric activity increases (i.e., low residence time) enzyme leakage increases which in turn decrease entrapment efficiency.

### 5.3. Kinetic Parameters

**Figure 5:**
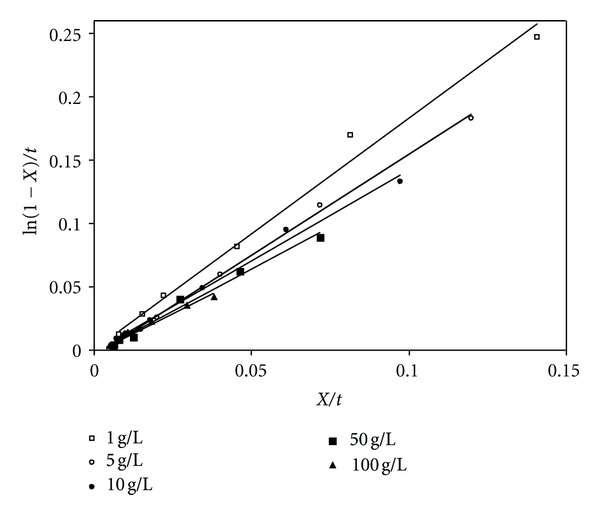
Linear plots of *X*/*t* versus ln⁡(1 − *X*)/*t* for immobilized *α*-amylase enzyme in fixed bed reactor (pH = 5.5, *T* = 50°C).

**Figure 6:**
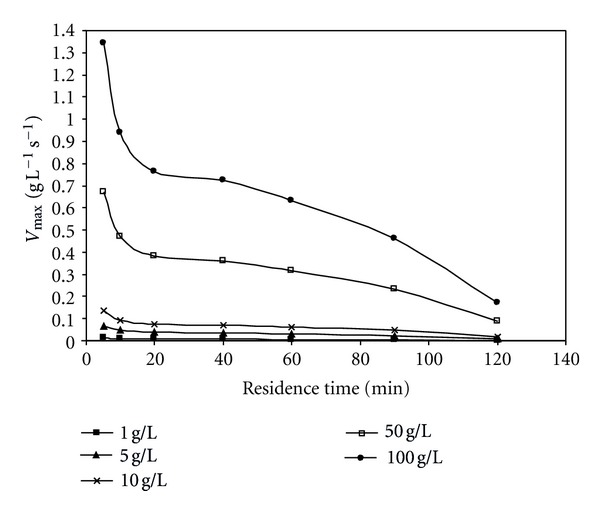
Effect of residence time on *V*_max⁡_ value at different initial substrate concentrations (pH = 5.5, *T* = 50°C).

**Figure 7:**
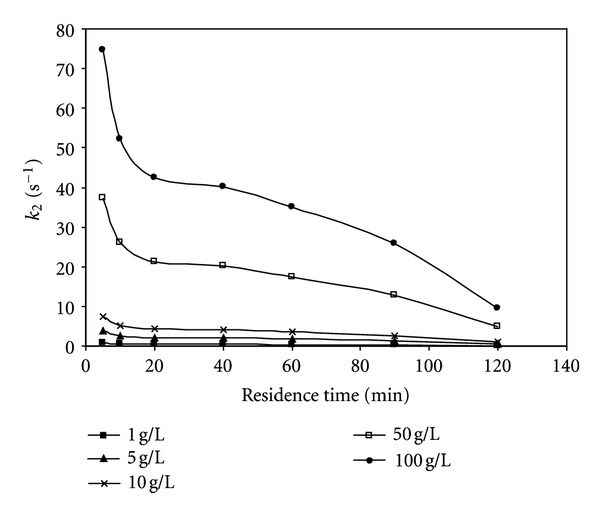
Effect of residence time on *k*_2_ value at different initial substrate concentrations (pH = 5.5, *T* = 50°C).

**Figure 8:**
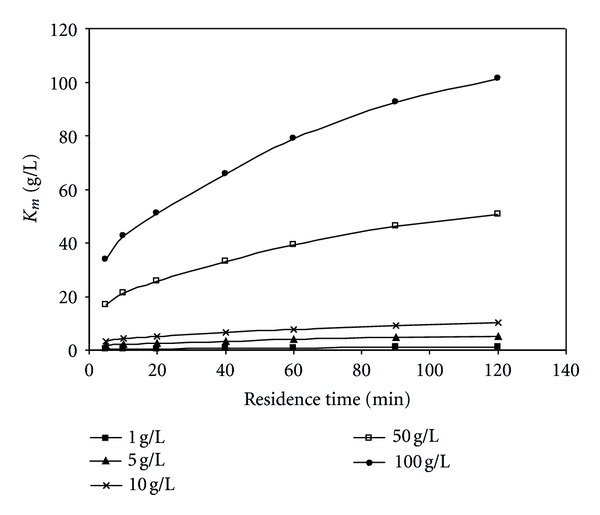
Effect of residence time on *K*_*m*_ value at different initial substrate concentrations (pH = 5.5, *T* = 50°C).

Kinetic parameters, Michaelis-Menten constant maximum activity *V*_max⁡_, *k*_2_, and *K*_*m*_ were determined at different substrate flow rates (i.e., residence times) and initial concentrations using (4) and (10). These values are estimated from the slope and intercept of the straight lines shown in Figure 5. The values of *V*_max⁡_, *k*_2_, and *K*_*m*_ at different residence times were listed in Table 1. These values were drawn against residence time and the relation between them can be shown in Figures 6, 7, and 8. As the residence time increases (i.e., substrate flow rate decreases), *V*_max⁡_ value decreases and it also increases with increasing initial substrate concentration. The *k*_2_ (which is named molecular activity, or turnover number of an enzyme, and is the number of substrate molecules converted to product by an enzyme molecule per unit time when the enzyme is fully saturated with substrate) value also decreases with increasing contact time and increases the initial substrate concentration because it is direct proportional to *V*_max⁡_ value according to (4). These observations can be attributed to the reason that with increasing residence time there is an increasing amount of substrate supplied to the enzyme and this phenomenon is not expected to continue for long where saturation has to be reached at some sites in the bead.

It can also be observed that *K*_*m*_ value increases with increasing residence time and initial substrate concentration. Thus, the lower the value of *K*_*m*_, the higher the affinity of enzyme for substrate. The velocity of the enzyme-catalyzed reaction is limited by the rate of breakdown of the ES complex. Then with increasing *K*_*m*_ value more products are formed. 

### 5.4. Simulation of Hydrolysis Reaction within the Hydrogel Bead

The solution for (17) was achieved using FINITE ELEMENTS in MATLAB V. 2008A software package.

**Figure 9:**
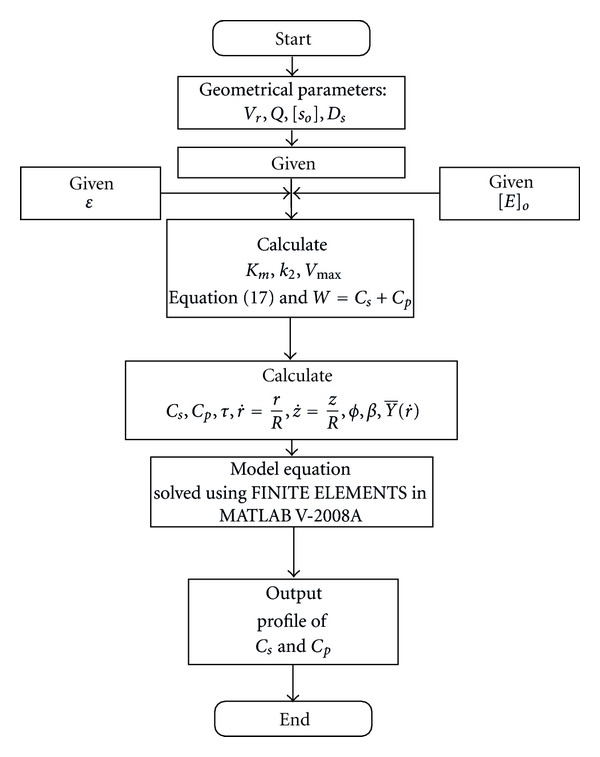
Algorithm to simulate substrate and product concentration profiles in a fixed bed reactor.

Figure 9 represent the algorithm for the computer simulation which is used to simulate substrate and product concentrations. 

The effective diffusivity of substrate was calculated at the proposed conditions of the present work and according to the references [33, 34]. The details of the method and equations are illustrated in these references. This value is equal to 7.8 × 10^−8^ cm^2^/min. 

**Figure 10:**
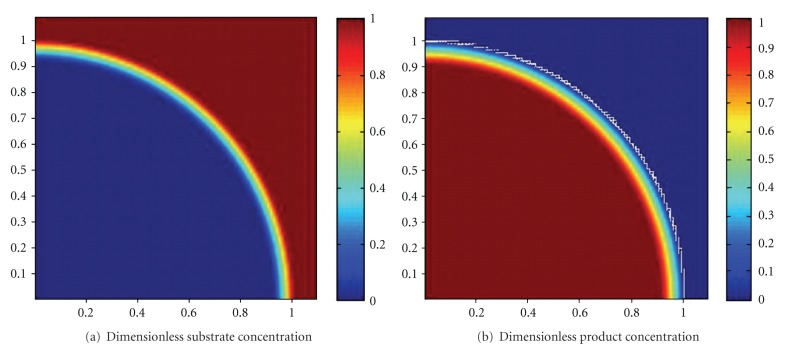
Dimensionless substrate and product concentration profiles in 3D (at *ϕ* = 47.6 and *t* = 5 min). The completely dark red area represents the region in which the substrate or product is at its maximum value (i.e., equilibrium value). The *x*-axis represents the dimensionless radius and the *y*-axis represents the *z*-axis.

**Figure 11:**
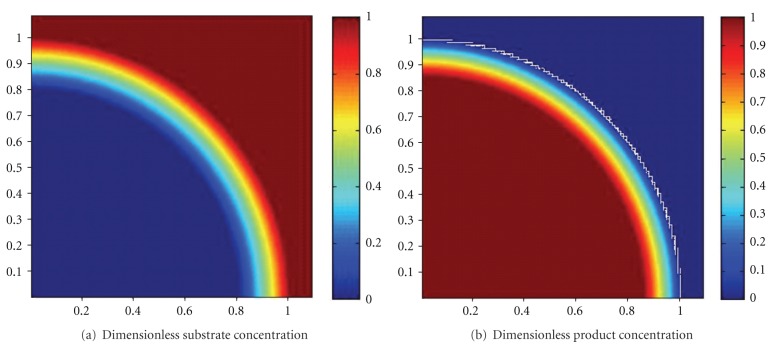
Dimensionless substrate and product concentration profiles in 3D (at *ϕ* = 35.4 and *t* = 10 min). The completely dark red area represents the region in which the substrate or product is at its maximum value (i.e., equilibrium value). The *x*-axis represents the dimensionless radius and the *y*-axis represents the *z*-axis.

**Figure 12:**
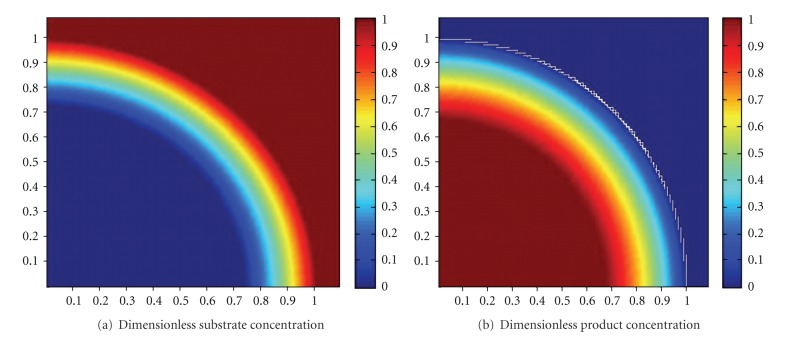
Dimensionless substrate and product concentration profiles in 3D (at *ϕ* = 29.2 and *t* = 20 min). The completely dark red area represents the region in which the substrate or product is at its maximum value (i.e., equilibrium value). The *x*-axis represents the dimensionless radius and the *y*-axis represents the *z*-axis.

**Figure 13:**
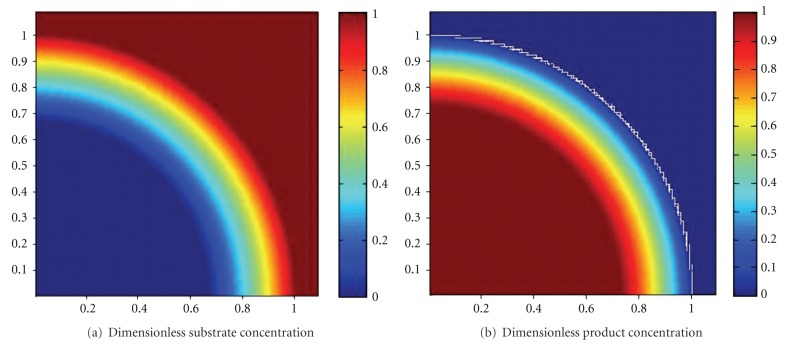
Dimensionless substrate and product concentration profiles in 3D (at *ϕ* = 25 and *t* = 40 min). The completely dark red area represents the region in which the substrate or product is at its maximum value (i.e., equilibrium value). The *x*-axis represents the dimensionless radius and the *y*-axis represents the *z*-axis.

**Figure 14:**
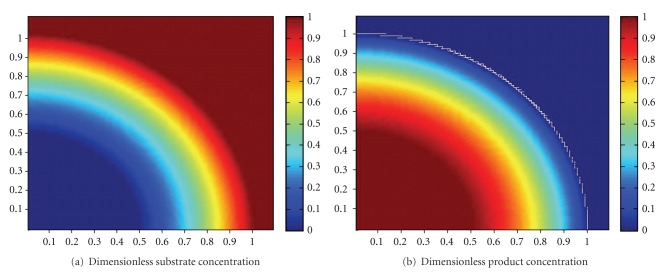
Dimensionless substrate and product concentration profiles in 3D (at *ϕ* = 21 and *t* = 60 min). The completely dark red area represents the region in which substrate or product at its maximum value (i.e. equilibrium value). *x*-axis represent dimensionless radius and *y*-axis represent *z*-axis.

**Figure 15:**
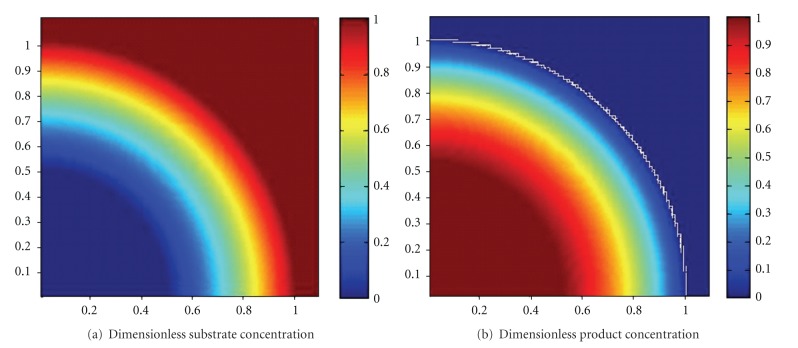
Dimensionless substrate and product concentration profiles in 3D (at *ϕ* = 16.8 and *t* = 90 min). The completely dark red area represents the region in which the substrate or product is at its maximum value (i.e., equilibrium value). The *x*-axis represents the dimensionless radius and the *y*-axis represents the *z*-axis.

**Figure 16:**
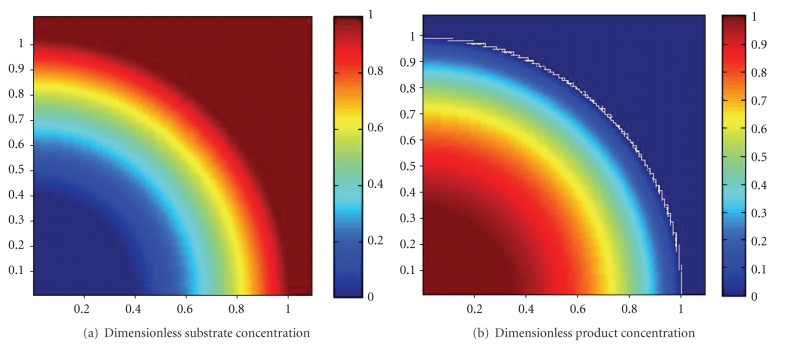
Dimensionless substrate and product concentration profiles in 3D (at *ϕ* = 9.8 and *t* = 120 min). The completely dark red area represents the region in which the substrate or product is at its maximum value (i.e., equilibrium value). The *x*-axis represents the dimensionless radius and the *y*-axis represents the *z*-axis.

The simulated dimensionless substrate and product concentration profiles are shown in Figures 10, 11, 12, 13, 14, 15, and 16. The color-scale map was used to study the substrate and product concentration profiles within the bead. It was assumed that two phases exist: solid bead phase and bulk liquid phase. At low substrate residence time (i.e., high substrate flow rate), the substrate drops rapidly only near the interface between the bead phase and the bulk liquid phase, and in this case the substrate reacts in a fast manner and it will never diffuse into the internal part of the bead and this is can be shown very well in Figure 10(a); on the other hand, the product is formed near the interface and diffuses at a very slow rate (as shown in Figure 10(b)). As the residence time increases (as shown in Figure 10(a) and Figure 16(a)) the reaction region increases and the substrate diffuses into the internal part of the bead. On the other hand, the product formed in a very slow manner and diffused at a slow rate so its concentration remains nearly low (as shown in Figure 10(b) to Figure 16(b)). According to assumption IV listed above, the system is at a steady state. Thus the composition and mass must be unchanged; substrate cannot accumulate in the shell. 

**Figure 17:**
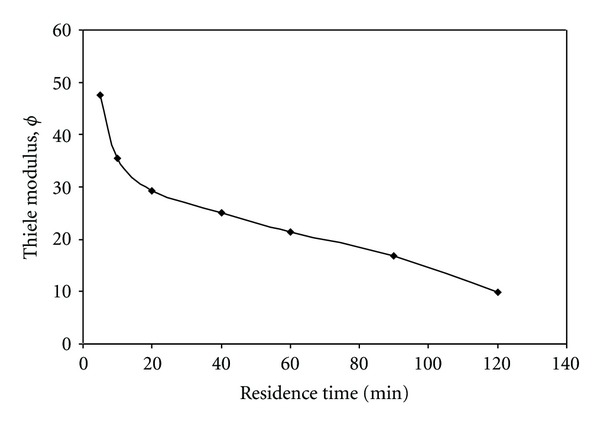
Effect of residence time on Thiele modulus (*ϕ*) value (pH = 5.5, *T* = 50°C).

**Figure 18:**
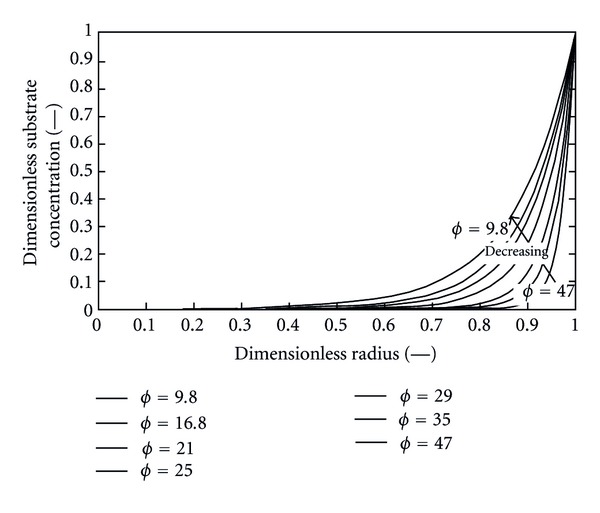
Dimensionless substrate concentration profile within the bead at steady state at different values of *ϕ* in 2D.

The relation between Thiele modulus (*ϕ*) and the residence time can be well implemented by Figure 17. The *ϕ* is a measure of whether the process is reaction rate controlled at low *ϕ* or diffusion rate controlled at high*ϕ*. It can be observed that *ϕ* increases with decreasing residence time. As *ϕ* increases, this means that mass transfer is much slower than the reaction; it is possible that all substrate entering the particle will be consumed before reaching the center of the bead. In this case, the concentration drops rapidly within the solid as illustrated by Figure 18. The active sites occupied by the immobilized enzyme near the center are starved of substrate and the core of the bead becomes inactive. At low *ϕ* value the concentration of substrate within the bead is naturally higher or lower than in the liquid phase. It can be stated from Figure 17 that starch hydrolysis at the proposed conditions is reaction rate controlled. Its reaction rate controllability decreases as the value of *ϕ* decreases.

## 6. Conclusions

In the present study, the performance of starch hydrolysis using *α*-amylase immobilized enzyme in a fixed bed reactor at steady state conditions was presented. The numerical solution of mass balances differential equations expressed in terms of a set of dimensionless parameters with the assumed initial and boundary conditions gave the following conclusions.

- The system performance is strongly affected by the substrate flow rate (i.e., residence time) and initial concentration.
- The results of reactor performance experiments demonstrated that the immobilized amylase retained 95% of activity after 20 h of continuous operation at 0.21 ml/min feed flow rate while at 5.04 mL/min the retained activity is 70%. Hence, it could be concluded that the immobilization of amylase in calcium alginate is very useful for continuous starch hydrolysis.
- The determined Thiele modulus (*ϕ*) values indicated that the reaction rate was controlled by reaction rate within the calcium alginate hydrogel beads.
- A decrease in *ϕ* values determines an improvement of substrate conversion.
- The simulation, which has been performed with experimental data, gave satisfactory results for the substrate and product concentration profiles.
- The simulation results comprise a valuable tool for immobilized enzyme reactor design by providing a quantitative relation of enzyme performance with operational variables like substrate flow rate, initial concentration, conversion, and particle size.

## Figures and Tables

**Table 1:** *K*_*m*_, *V*_max⁡_, and *k*_2_ values of starch hydrolysis using immobilized amylase enzyme in fixed bed reactor at different values of initial substrate concentration and residence time (pH = 5.5, *T* = 50°C).

[*S*] g/L	*K*_*m*_ (g/L)						
	Residence time, min						
	5	10	20	40	60	90	120
1	0.340	0.428	0.510	0.658	0.790	0.926	1.010
5	1.690	2.137	2.550	3.290	3.940	4.630	5.060
10	3.380	4.270	5.100	6.580	7.870	9.260	10.13
50	16.89	21.37	25.51	32.90	39.37	46.30	50.66
100	33.78	42.74	51.00	65.79	78.74	92.60	101.3

	*V*_max⁡_ (g/L·s)						

1	0.013	0.009	0.008	0.007	0.006	0.005	0.002
5	0.067	0.047	0.038	0.036	0.181	0.023	0.009
10	0.134	0.094	0.077	0.072	0.362	0.046	0.017
50	0.672	0.470	0.383	0.362	1.811	0.232	0.086
100	1.344	0.940	0.765	0.724	3.622	0.463	0.172

	*k*_2_ (s^−1^)						

1	0.747	0.522	0.425	0.402	0.350	0.257	0.096
5	3.734	2.611	2.126	2.010	10.06	1.286	0.478
10	7.467	5.223	4.252	4.020	20.12	2.572	0.957
50	37.34	26.12	21.26	20.10	100.6	12.86	4.784
100	74.68	52.23	42.52	40.20	201.2	25.72	9.569

## Nomenclature

*C*: Dimensionless concentration (—)
*D*: Effective diffusivity (cm^2^/min)
*E*: Enzyme concentration (g/L)
[*E*]_0_: Initial enzyme concentration (g/L)
*ES*: Enzyme-substrate complex (—)
*k*: Rate constant (*s* ^−1^)
*K*_*m*_: Michaelis constant (g/L)
*P*: Product concentration (g/L)
*Q*: Substrate flowrate, (cm^3^/min)
*r*: Radius of immobilized enzyme bead (cm)
*R*: Radius of immobilized enzyme bead (cm)
$\dot{r}$: Dimensionless radius (—)
*S*: Substrate concentration (g/L)
*t*: time (min)
*v*: Rate of reactant decomposition (gL^−1^ *s* ^−1^)
*V*: Rate of reaction in (2) (gL^−1^ *s* ^−1^)
*V*: Volume (cm^3^)
*W*: Dimensionless concentration (—)
*X*: Conversion (%)
$\dot{z}$: Diminsionless hight (—).

### Subscript

en: Entrapped enzyme amount in the beads
un: Unentrapped enzyme amount in the reactor outlet solution
1: Forward reaction between enzyme and substrate
-1: Backward reaction
2: ES complex dissociation
*b*: Bead in equation *ε* = *V*_*b*_/*V*_*R*_
*b*: Bulk solution
max⁡: Maximum value
0: Initial value (—)
*p*: Product
*R*: Reactor
*s*: Substrate
*t*: Concentration at time *t*.

### Symbols

*∅*: Thiel's modulus, $\emptyset =\left(R/4\right)\sqrt{V_{\max}/D_{s}k_{m}}$(—)
*τ*: Dimensionless time (—)
*β*: Dimensionless ratio (—)
*ε*: Voidage (—).

## References

[1] Valencia P, Wilson L, Aguirre C, Illanes A (2010). Evaluation of the incidence of diffusional restrictions on the enzymatic reactions of hydrolysis of penicillin G and synthesis of cephalexin. *Enzyme and Microbial Technology*.

[2] Djabali D, Belhaneche N, Nadjemi B, Dulong V, Picton L (2009). Relationship between potato starch isolation methods and kinetic parameters of hydrolysis by free and immobilised *α*-amylase on alginate (from Laminaria digitata algae). *Journal of Food Composition and Analysis*.

[3] Gangadharan D, Nampoothiri KM, Sivaramakrishnan S, Pandey A (2009). Immobilized bacterial *α*-amylase for effective hydrolysis of raw and soluble starch. *Food Research International*.

[4] Illanes A (2011). Immobilized biocatalysts. *Comprehensive Biotechnology*.

[5] Doran PM (1995). Heterogeneous reactions. *Bioprocess Engineering Principles*.

[6] Gülay S (2007). *Immobilization of thermophilic recombinant esterase enzyme by entrapment in coated Ca-alginate beads [M.S. thesis]*.

[7] Konsoula Z, Liakopoulou-Kyriakides M (2006). Thermostable *α*-amylase production by *Bacillus subtilis* entrapped in calcium alginate gel capsules. *Enzyme and Microbial Technology*.

[8] Talekar S, Chavare S (2012). Optimization of immobilization of *α*-amylase in alginate gel and its comparative biochemical studies with free *α*-amylase. *Recent Research in Science and Technology*.

[9] Meyer LN (2007). *Effect of immobilization method on activity of alpha-amylase [thesis]*.

[10] Demirkan E, Dincbas S, Sevinc N, Ertan F (2011). Immobilization of *B. amyloliquefaciensα*-amylase and comparison of some of its enzymatic properties with the free form. *Romanian Biotechnological Letters*.

[11] Riaz A, Qader SAU, Anwar A, Iqbal S (2009). Immobilization of a thermostable A-amylase on calcium alginate beads from *Bacillus subtilis* KIBGE-HAR. *Australian Journal of Basic and Applied Sciences*.

[12] Dey G, Singh B, Banerjee R (2003). Immobilization of *α*-amylase produced by *Bacillus circulans* GRS 313. *Brazilian Archives of Biology and Technology*.

[13] Dincbas S, Demirkan E (2010). Comparison of hydrolysis abilities onto soluble and commercial raw starches of immobilized and free *B. amyloliquefaciensα*-amylase. *Journal of Biological & Environmental Sciences*.

[14] Anwar A, Qader SAU, Raiz A, Iqbal S, Azhar A (2009). Calcium Alginate: a support material for immobilization of proteases from newly isolated strain of *Bacillus subtilis* KIBGE-HAS. *World Applied Sciences Journal*.

[15] Park D, Haam S, Jang K, Ahn IS, Kim WS (2005). Immobilization of starch-converting enzymes on surface-modified carriers using single and co-immobilized systems: properties and application to starch hydrolysis. *Process Biochemistry*.

[16] Horta A, Álvarez JR, Luque S (2007). Analysis of the transient response of a CSTR containing immobilized enzyme particles. Part I. Model development and analysis of the influence of operating conditions and process parameters. *Biochemical Engineering Journal*.

[17] Jeison D, Ruiz G, Acevedo F, Illanes A (2003). Simulation of the effect of intrinsic reaction kinetics and particle size on the behaviour of immobilised enzymes under internal diffusional restrictions and steady state operation. *Process Biochemistry*.

[18] Dadvar M, Sahimi M (2002). Pore network model of deactivation of immobilized glucose isomerase in packed-bed reactors II: Three-dimensional simulation at the particle level. *Chemical Engineering Science*.

[19] Dadvar M, Sohrabi M, Sahimi M (2001). Pore network model of deactivation of immobolized glucose isomerase in packed-bed reactors I: Two-dimensional simulations at the particle level. *Chemical Engineering Science*.

[20] Loghambal S, Rajendran L (2011). Mathematical modeling in amperometric oxidase enzyme-membrane electrodes. *Journal of Membrane Science*.

[21] Al-Muftah AE, Abu-Reesh IM (2005). Effects of internal mass transfer and product inhibition on a simulated immobilized enzyme-catalyzed reactor for lactose hydrolysis. *Biochemical Engineering Journal*.

[22] Bhatia S, Long WS, Kamaruddin AH (2004). Enzymatic membrane reactor for the kinetic resolution of racemic ibuprofen ester: modeling and experimental studies. *Chemical Engineering Science*.

[23] Zhang H, Jin Z (2011). Preparation of products rich in resistant starch from maize starch by an enzymatic method. *Carbohydrate Polymers*.

[24] Varatharajan V, Hoover R, Li J (2011). Impact of structural changes due to heat-moisture treatment at different temperatures on the susceptibility of normal and waxy potato starches towards hydrolysis by porcine pancreatic alpha amylase. *Food Research International*.

[25] Mukherjee A, Ghosh AK, Sengupta S (2010). Purification and characterization of a thiol amylase over produced by a non-cereal non-leguminous plant, *Tinospora cordifolia*. *Carbohydrate Research*.

[26] Kumar RSS, Vishwanath KS, Singh SA, Rao AGA (2006). Entrapment of *α*-amylase in alginate beads: single step protocol for purification and thermal stabilization. *Process Biochemistry*.

[27] Konsoula Z, Liakopoulou-Kyriakides M (2006). Starch hydrolysis by the action of an entrapped in alginate capsules *α*-amylase from *Bacillus subtilis*. *Process Biochemistry*.

[28] Marangoni AG (2003). *Enzyme Kinetics a Modern Approach*.

[29] Lee JM (2011). *Biochemical Engineering*.

[30] Arica MY, Hasirci V, Alaeddinoğlu NG (1995). Covalent immobilization of *α*-amylase onto pHEMA microspheres: preparation and application to fixed bed reactor. *Biomaterials*.

[31] Bernfeld P, Colowick SP, Kaplan NO (1955). Amylases *α* and *β*. *Methods in Enzymology*.

[32] http://www.sigmaaldrich.com/sigma-aldrich/technical-documents/protocols/biology/enzymatic-assay-of-a-amylase.html.

[33] Grunwald P, Hansen K, Gunber W (1997). The determination of effective diffusion coefficients in a polysaccharide matrix used for the immobilization of biocatalysts. *Solid State Ionics*.

[34] Coulson J, Richardson MJF, Backhurst JR, Marker JH (1999). *Chemical Engineering*.

